# Bell’s Palsy After Second Dose of Moderna COVID-19 Vaccine: Coincidence or Causation?

**DOI:** 10.15388/Amed.2021.28.2.7

**Published:** 2021-08-04

**Authors:** Sohil Pothiawala

**Affiliations:** Department of Emergency Medicine, Woodlands Health Campus, Singapore **ORCID id:** https://orcid.org/0000-0002-4789-4326

**Keywords:** Bell’s palsy, mRNA, Moderna, vaccine

## Abstract

US Food and Drug Administration (FDA) recommended enhanced safety surveillance to monitor for cases of Bell’s palsy following Moderna vaccine administration in larger populations. The author reports a patient who developed right sided Bell’s palsy 2 weeks after administration of the second dose of Moderna COVID-19 vaccine. Considering this development of symptoms 2 weeks after the second dose of Moderna vaccine administration, there remains a possibility of a causal association. As more people get vaccinated, more information may be available in the future to establish association. Physicians need to maintain enhanced safety surveillance to monitor for cases of Bell’s palsy following mRNA vaccine administration.

## Abstract (lt)

**Belo paralyžius pasiskiepijus antrąja „Moderna“ COVID-19 doze. Atsitiktinumas ar priežastingumas?:**

**Santrauka. **JAV maisto ir vaistų administracija (FDA) rekomendavo atidžiau stebėti „Moderna“ paskiepytus gyventojus Belo paralyžiaus atvejams nustatyti. Autorius praneša apie atvejį, kai pacientui išsivystė dešiniosios pusės Belo paralyžius praėjus dviem savaitėms po skiepijimosi antrąja „Moderna“ COVID-19 doze. Galimas priežastinis ryšys atsižvelgiant į atsiradusius simptomus praėjus dviem savaitėms po skiepijimosi „Moderna“. Paskiepijus didesnį kiekį žmonių ateityje galima turėti daugiau informacijos šiam ryšiui nustatyti. Gydytojai turėtų atidžiau stebėti pacientus dėl galimų Belo paralyžiaus atvejų pasiskiepijus mRNA.

**Raktažodžiai:** Belo paralyžius, mRNA, „Moderna“, skiepas

## Introduction

Bell’s palsy, an idiopathic 7^th^ cranial nerve palsy, occurs in 12–25 per 100,000 people in the general population. There has been reported association of facial nerve palsy after influenza vaccination, and is thought to be either immune mediated or induced by viral reactivation [1].

Two mRNA coronavirus disease 2019 (COVID-19) vaccines have received emergency use authorization by the US Food and Drug Administration (FDA), and have been deployed for public vaccination in many countries around the world. The rate of facial paralysis after mRNA COVID-19 vaccination is not high when compared to other viral vaccines [2]. The author reports a patient who developed right sided Bell’s palsy 2 weeks after administration of the second dose of Moderna COVID-19 vaccine.

## Case Report

**Fig 1.**
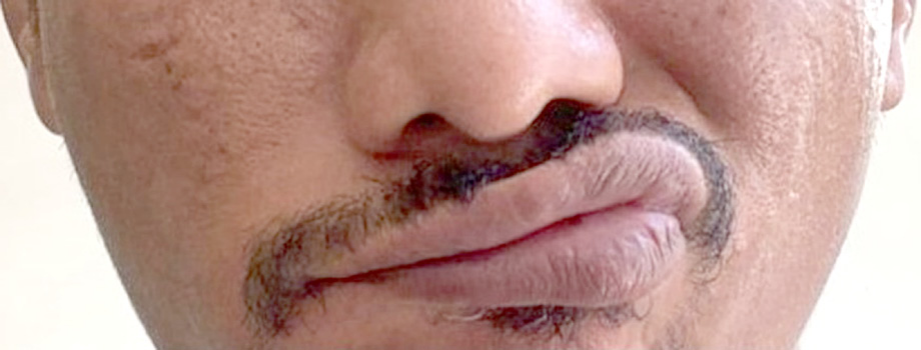
Photo of the patient with right sided facial droop and loss of right naso-labial fold

**Fig 2.**
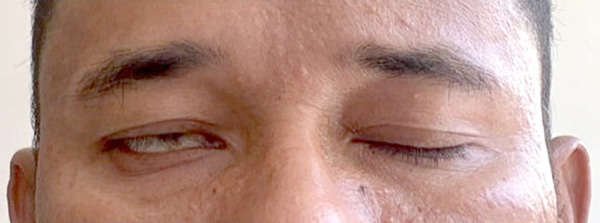
Inability of the patient to close right eye completely

A 46-year-old male with no past medical history received his second dose of Moderna COVID-19 vaccine. 10 days following the vaccination, he noticed loss of taste sensation along with tinnitus in the right ear. On the 13^th^ day after vaccination, he discovered that he developed right sided facial droop and loss of right naso-labial fold (Fig 1), as well as inability to close his right eye completely (Fig 2). 

He denied any other symptoms. He went to the emergency department and his vital signs were stable. His neurological examination was normal except for isolated right lower motor neuron 7^th^ nerve palsy. There was no history of any preceding infection, including recent SARS-CoV-2 infection, and there was no evidence of rash suggestive of Herpes Zoster. His gait was normal and he was diagnosed to have right Bell’s palsy. Bell’s palsy is a clinical diagnosis and there is no specific laboratory test to confirm the diagnosis. Diagnostic tests are generally done when the clinical presentation is atypical, and can be done to exclude neuropathies, brain tumor or infections like Lyme disease, which is not common in our geographical location. As our patient presented with signs and symptoms typical of Bell’s palsy, further tests were not performed. He was commenced on antiviral medication acyclovir and prednisone. He was discharged with an outpatient follow-up with otorhinolaryngologist. 

## Discussion

Combined data of 40000 recruited participants over 2 months from the Pfizer-BioNTech and Moderna COVID-19 vaccine trials reported seven cases of Bell’s palsy in the vaccine arm compared with one case in the placebo arm. They concluded that considering the rate of Bell’s palsy in the general population, a causal relationship with the vaccine could not be established. But, this data suggests that the observed incidence of Bell’s palsy in the clinical trial vaccination arm is about 3.5–7 times higher than the rate in the general population [3].

In the Moderna COVID-19 vaccine clinical trial, there were three reports of Bell’s palsy in the vaccine group (one being a serious adverse event) compared to one in the placebo group. This occurred on days 22, 28, and 32 after the second dose in the three vaccine recipients. The fact sheet did not indicate any health data of the participants, or whether if any of these four patients in their trial had a previous history of Bell’s palsy [4]. But after deployment of the Moderna vaccine for public vaccination, FDA suggested that a causal relationship between the COVID-19 vaccines and Bell’s palsy could not be excluded. Hence, they recommended enhanced safety surveillance to monitor for cases of Bell’s palsy following vaccine administration in larger populations [5].

Considering development of symptoms of Bell’s palsy in our patient about 2 weeks after the second dose of Moderna vaccine administration, there remains a possibility of a causal association. Physicians need to be vigilant of early occurrence of this adverse event in response to the Moderna vaccine, as compared to the average 3 week-delayed onset of Bell’s palsy documented in the clinical trial. 

## Conclusion

Considering the previous association between influenza vaccine and Bell’s palsy, the time of onset of Bell’s palsy in our patient suggests that it could be related to Moderna vaccine. Though surveillance is important to recognize and report potential Moderna vaccine-related Bell’s palsy, most cases of Bell’s palsy are self-limiting and resolve over time. Presently, the benefits of the COVID-19 vaccination outweigh the risks in this current pandemic. But, as more people get vaccinated, more information may be available in the future to establish a potential causal association.

## Meeting

This manuscript has not been presented in abstract form at any meeting.

## References

[ref1] Kamath A, Maity N, Nayak MA. Facial paralysis following influenza vaccination: a disproportionality analysis using the Vaccine Adverse Event Reporting System Database. Clin Drug Investig. 2020; 40(9):883–889. doi: 10.1007/s40261-020-00952-0 PMC7371962 32696320

[ref2] Renoud L, Khouri C, Revol B, Lepelley M, Perez J, Roustit M, et al. Association of facial paralysis with mRNA COVID-19 vaccines: A disproportionality analysis using the World Health Organization pharmacovigilance database. JAMA Intern Med 2021 Apr 27; e212219. doi: 10.1001/jamainternmed.2021.2219. [Online ahead of print] PMC8080152 33904857

[ref3] Ozonoff A, Nanishi E, Levy O. Bell’s palsy and SARS-CoV-2 vaccines. Lancet Infect Dis 2021; 21(4):450–452. doi: 10.1016/S1473-3099(21)00076-1 33639103 PMC7906673

[ref4] Fact sheet for healthcare providers administering vaccine (vaccination providers). Emergency use authorization (EUA) of the Moderna COVID-19 vaccine to prevent coronavirus disease 2019 (COVID-19) dated March 31, 2021. https://www.modernatx.com/covid19vaccine-eua/eua-fact-sheet-providers.pdf. Accessed on 7th June 2021.

[ref5] Repajic M, Lai XL, Xu P, Liu A. Bell’s Palsy after second dose of Pfizer COVID-19 vaccination in a patient with history of recurrent Bell’s palsy. Brain Behav Immun Health. 2021 May; 13: 100217. doi: 10.1016/j.bbih.2021.100217 33594349 PMC7874945

